# GLP-1 Targeting Agents Impair Chemoimmunotherapy Effectiveness in Triple-Negative Breast Cancer

**DOI:** 10.21203/rs.3.rs-8380296/v1

**Published:** 2026-01-09

**Authors:** Bethania Santos, Maycon Marção, Ishrat Durdana, Brian Lee, Lavanya Vumma, Felipe Segato-Dezem, Hannah Chasteen, Song Zhang, Cheryl Lewis, Yan Peng, Alexis LeVee, Megan Wong, Joanne Mortimer, Heather McArthur, Philipp E. Scherer, Jasmine T. Plummer, Joshua Gruber

**Affiliations:** 1. Internal Medicine, Cecil H. and Ida Green Center for Reproductive Biology Sciences, Harold C. Simmons Comprehensive Cancer Center, UT Southwestern Medical Center, Dallas, TX, USA; 2. Center for Spatial Omics, St Jude Children’s Research Hospital, Tennessee, USA; 3. Department of Developmental Biology, St Jude Children’s Research Hospital, Tennessee, USA; 4. O’Donnell SPH-Health Data Sci Biostats, Dallas, TX, USA; 5. Pathology Department & Harold C. Simmons Comprehensive Cancer Center, UT Southwestern Medical Center, Dallas, TX, USA; 6. City of Hope Comprehensive Cancer Center, Duarte, CA, USA; 7. Touchstone Diabetes Center, UT Southwestern Medical Center, Dallas, TX.

## Abstract

Activation of glucagon-like peptide-1 receptor (GLP-1R) could affect cancer treatment responses through direct action in tumor or immune cells. However, the field lacks a comprehensive assessment of GLP-1R expression and activity across human tumors. Herein, we report detection GLP-1R across multiple human tumor types and focus on triple-negative breast cancer (TNBC) for deeper analysis. In TNBC, GLP-1R is present in immune and tumor cell compartments. GLP-1 treatment of cancer cells activated survival pathways, drove proliferation, induced paclitaxel resistance and dampened cytokine secretion, effects that required expression of GLP-1R. Spatial transcriptomics of human tumors revealed that GLP-1 exposure remodeled the tumor microenvironment, promoted a mesenchymal transition in malignant cells and disrupted productive macrophage inflammation in tumor-proximate niches. Patients taking GLP-1 drugs during neoadjuvant chemotherapy experienced reduced pathological complete response rates (pCR: 30.8%) compared to controls (65%, p<0.001). Thus, GLP-1-exposure acts on tumor and immune cells to impair chemoimmunotherapy efficacy in TNBC.

## Introduction

Triple-negative breast cancer (TNBC) is an aggressive subtype of breast cancer, characterized by limited treatment options and poorer outcomes compared to other breast cancer subtypes. The recent incorporation of immune checkpoint inhibitors into neoadjuvant regimens, including the KEYNOTE-522 protocol (pembrolizumab combined with multi-agent chemotherapy [carboplatin, paclitaxel, doxorubicin, cyclophosphamide]), has improved pathological complete response (pCR) rates and survival.^1,2^ However, significant variability in treatment response persists, prompting an investigation into factors that may modulate the efficacy of chemoimmunotherapy, including comorbid conditions and concomitant medications.^3^

Glucagon-like peptide-1 receptor agonists (GLP-1RAs), including exenatide, liraglutide, tirzepatide, and semaglutide, have become key treatments for type 2 diabetes mellitus (T2DM) and obesity, diseases frequently co-occurring with TNBC.^4,5,6,7,8^ These incretin mimetics exert their glucose-lowering effects primarily through GLP-1 receptor (GLP-1R) activation but overcome the pharmacokinetic limitation of endogenous GLP-1, which is rapidly degraded by dipeptidyl peptidase 4 (DPP4) with a plasma half-life of approximately 2 minutes. The development of DPP4-resistant GLP-1RAs with extended half-lives has enabled sustained receptor activation.^9^ Similarly, DPP4 inhibitors (sitagliptin, vildagliptin, saxagliptin, linagliptin, and alogliptin) prolong endogenous GLP-1 activity and are commonly used as monotherapy or in combination with other antihyperglycemic agents like metformin.^10^

Despite the widespread use of GLP-1RAs and DPP4 inhibitors (DPP4i), their impact on cancer outcomes remains poorly characterized. Prior epidemiological studies have yielded conflicting results, with some suggesting effects on cancer risk and others reporting potential associations with worse outcomes in certain malignancies.^11,12,13,14^ Preclinical studies indicate GLP-1R signaling on immune cells can drive an immunosuppressive phenotype^15,16,17^ and has been linked to pro-survival pathways in other cellular contexts^18,19^. However, this could be balanced by improved organismal metabolism due to weight loss or other systemic effects that could positively affect cancer outcomes. This uncertainty raises an important question: could pharmacologic activation of GLP-1R alter cancer treatment response?

To test this, we integrated a spatial transcriptomics and functional cell biological assays with multicenter clinical study of breast cancer patients receiving the KEYNOTE-522 chemoimmunotherapy regimen. These findings demonstrate that GLP-1 exposure drives treatment resistance through complementary pathways: intrinsically by activating pro-survival signaling and EMT, and extrinsically by altering tumor-macrophage niches. This investigation addresses an urgent clinical dilemma at the intersection of two prevalent diseases, with direct implications for the management of patients with TNBC and cardiometabolic comorbidities.

## Methods

### Cell biology & Signaling of GLP-1 in TNBC cell lines

#### Cell Lines:

All cell lines were cultured in a humidified incubator at 37°C, with 5% CO_2_ in RPMI media supplemented with 10% FBS and 1% penicillin/streptomycin. Cell lines HCC1395 (human female, RRID: CVCL 1249) and HCC1187 (human female, RRID: CVCL 1247) were gifts from John Minna. T47D cells (human female, RRID: CVCL 0I95) were a gift from Carlos Arteaga. Cell lines were authenticated by morphology and STR analysis. Cell lines are tested quarterly for mycoplasma with PCR assay (last test July 2025). Experiments were performed with passage 1–3 cell line stocks that were maintained in culture for one month or less. Cell growth rates were determined by Incucyte to obtain linear growth rates (confluence measurement) of at least triplicate wells in 96-well plates over 24–72 hours, which were then fit by linear regression in Graphpad Prism (version 10) to derive growth rates (m). Alternatively, Cell Titer Glo (Promega G9243) was used as an endpoint assay according to manufacturer’s instructions.

#### Cytokine analysis:

HCC1395 cells were cultured in the presence of semaglutide (5 or 10 μM) versus vehicle control for 72 h then media was diluted 1:2 and applied to R&D Luminex custom cytokine panel according to manufacturer’s instructions.

#### Protein electrophoresis and immunoblotting:

Protein extracts were made by sonication in RIPA buffer and quantitated by BCA assay and diluted to equal concentrations. Polyacrylamide gel electrophoresis was performed with NuPAGE Novex gradient gels (Invitrogen NW04122BOX) followed by wet transfer to nitrocellulose membranes. Blocking was briefly performed with 5% non-fat milk and primary antibody was incubated overnight at 4°C, followed by incubation with HRP-conjugated secondary antibody (Cell Signaling, rabbit CST 7074s, mouse CST 7076s) at room temperature for 1 hour followed by washing, then developed with ECL femto (Thermo Fisher 34096). Primary antibodies included α-phospho-Akt S476 (Cell Signaling #4058, RRID:AB_331168), α-Akt (Cell Signaling #4685, RRID:AB_2225340), α-phospho-ERK (Cell Signaling #9101, RRID:AB_331646), α-ERK (Cell Signaling #4695, RRID:AB_390779), α-phospho-CREB Ser133 (Cell Signaling #9198, RRID:AB_ 2561044), α-CREB (Cell Signaling #9197, RRID:AB_ 331277), α-phospho-S6 Ser235/236 (Cell Signaling #2211, RRID:AB_ 331679), α-S6 (Cell Signaling #2317, RRID:AB_2238583).

### Immunohistochemical Analysis of GLP-1R Expression in TNBC Specimens

Archival formalin-fixed, paraffin-embedded (FFPE) primary tumor specimens were obtained from a previously reported cohort^20^ of female patients (≥18 years) with histologically confirmed triple-negative breast cancer (TNBC; ER/PR <10% and HER2-negative per ASCO/CAP guidelines). Cases were selected based on specimen availability and adequacy for IHC analysis from The UTSW Simmons Cancer Center Tissue Management Shared Resource and Parkland Health approved by UTSW IRB.

The cores were taken from areas in the paraffin block that were representative of the diagnostic tissue based on examination of the original H&E slides. Tissue blocks were sectioned (4 μM) onto standard microscope slides, baked at 60C for 30 minutes and deparaffinized. Antigen retrieval was performed using pH=9 EDTA buffer (95–100°C, 20 min) on a Leica Bond RX system. For antibody staining, tissue sections were incubated with a rabbit monoclonal anti-GLP-1 antibody (Abcam #ab254352 [RRID_3717471]; 1:50 dilution for 60 minutes; pre-adsorption controls confirmed specificity). The staining was visualized using the Bond Polymer Refine detection system without the Bond post-primary reagent.

GLP-1R expression was evaluated semi-quantitatively by an expert breast pathologist in both tumor cells and the tumor microenvironment (TME) (including stromal fibroblasts, endothelial cells, and immune populations) using a three-tiered scoring system: 0 (negative; no detectable staining), 1 (positive; weak-to-moderate cytoplasmic/membrane staining), or 2 (strong positive; intense, clustered, or diffuse membrane-specific reactivity).

### CosMx 6000-plex spatial transcriptomics

#### Patient material and Data collection

To characterize the spatial architecture and cellular context of GLP-1R expression in TNBC, we conducted high-plex spatial RNA profiling with the CosMx Spatial Molecular Imager platform in a clinically annotated cohort of 11 patients treated with neoadjuvant chemo-immunotherapy. The cohort was identified through institutional registries and pharmacy records and included 7 patients who received GLP-1 targeting agents and 4 controls on other antidiabetic medications (non-exposed group), balanced for age, body mass index, comorbidities, and tumor stage (Supplementary Table S1). A total of 16 FFPE tissue sections were analyzed, comprising paired pre- and post-treatment biopsies or pre-treatment samples from patients who achieved a complete pathological response. The study was approved by the UTSW Institutional Review Board.

#### CosMx Assay Preparation

Six breast tissue blocks were sectioned onto each standard microscope slide and baked vertically overnight at 37°C for 17 hours and 60°C for 3 hours. Slides were deparaffinized in two Xylene washes and rehydrated in 100% Ethanol. To prepare the tissue for probe attachment, slides underwent heat-induced epitope retrieval, proteinase K digestion, fiducial application, fixation with 10% Neutral Buffered Formalin, and NHS-acetate incubation. The denatured CosMx Human Discovery probe panel was applied to each slide and incubated overnight at 37°C for 17 hours.

After overnight probe hybridization, slides were washed in a 50:50 mixture of 100% formamide and 4X SSC at 37°C to remove off-target probes. Slides were stained with DAPI (nuclear stain), CD298/B2M (membrane stain), PanCK (epithelial cells), and CD45 (immune cells). After staining was completed, a CosMx flow cell was attached to each slide. Instrument buffer bottles, reagent tray, and slides were loaded onto the CosMx SMI and run using pre-bleaching profile Configuration C and cell segmentation profile Configuration A. Fields of view (FOVs) were selected to encompass each sample. Reporters were cycled over each FOV and imaged to determine which targets are present in each sample.

#### Data Processing and Preprocessing

Raw CosMx Spatial Molecular Imaging (SMI) data was first obtained as gene-by-cell count matrices per tissue section, following Bruker’s CosMx 6K RNA panel acquisition protocol. Each section was preprocessed individually. Initial quality control steps included filtering out low-quality cells (cells with fewer than a minimum number of detected transcripts or genes) and low-abundance genes (genes expressed in fewer than a defined threshold of cells). We retained only high-confidence transcripts with spatial coordinates for downstream analysis.

Next, spatial cell segmentation masks were imported to associate transcriptomic data with segmented cells, enabling spatially resolved gene expression quantification. Data normalization was performed using a log-normalization strategy to scale for sequencing depth across cells. Batch effects between sections and patients were assessed but no sections or sample batches needed correction. A unified spatial transcriptomics *Seurat (v5.1.0*) object was created to consolidate all sections, allowing for consistent data handling and cross-sample comparisons. Dimensionality reduction using PCA and UMAP was applied for visualization, and unsupervised clustering using shared nearest neighbor (SNN) modularity optimization was conducted to observe underlying structure in the data prior to annotation.

#### QC filtering

We filtered the data by integrating the AtoMx QC flag report with additional thresholds on cell-level transcriptomic metrics to ensure retention of high-quality segmented cells. Specifically, only cells with both *nCount* and *nFeature* values greater than 40 were retained for downstream analyses. nCount refers to the total number of unique molecular identifier (UMI) counts per cell, representing the total RNA molecules captured and sequenced. nFeature refers to the number of distinct genes detected per cell, serving as a measure of transcriptome complexity. These metrics were used to identify and remove low-quality cells such as empty droplets, dead cells, or technical artifacts. A visual inspection of the tissue sections was performed to identify samples where the RNA signal originated from only a portion of the tissue. One sample (#40119) displayed partial tissue coverage, with detectable RNA signal restricted to a portion of the section; in this sample, the affected region was manually cropped prior to analysis. No evidence of slide-level or section-specific batch effects was observed following QC filtering, as confirmed by visual inspection of feature distributions and dimensionality reduction embeddings.

#### Cell Type Annotation

To assign biological meaning to individual cell clusters, we performed cell type annotation on each section independently using *InsitutypeML*^21^, a supervised machine learning tool designed for cell type classification in spatial transcriptomic datasets. As a reference, we used the Bruker Breast Cancer CosMx 6K annotated cell atlas^22^, which contains pre-labeled cell types across breast cancer-associated lineages, including immune cells, stromal fibroblasts, and tumor epithelial subtypes.

Annotation performance was assessed using multiple complementary strategies. First, we visualized annotation results in each section using *flightpath_plot*, which overlays predicted cell types onto their spatial coordinates, enabling intuitive evaluation of spatial coherence and biological plausibility. Second, we transferred annotations to the unified Seurat object and projected them onto a UMAP embedding to assess global concordance of cell type distributions across sections.

To evaluate consistency in marker gene expression across independently annotated sections, we examined overlaps in key marker gene expression patterns. This ensured that equivalent cell types were consistently identified across spatial replicates. Lastly, we performed per-cell-type correlation analysis by comparing average expression profiles of each annotated cell type in the experimental dataset with their counterparts in the reference atlas. These gene-level correlation plots provide quantitative validation of annotation fidelity by highlighting agreement between observed and reference expression profiles for matched genes and cell types.

#### Cell Proportion Analysis

Cell type proportion analysis was conducted by annotating cell identities from transcript-based clustering and validated using canonical lineage markers. For each sample, the relative abundance of major cell populations (epithelial, immune, and stromal) was calculated as the fraction of each cell type among all segmented cells passing quality control. Cell type proportional analysis for GLP-1 exposed vs non-exposed was plotted using ggplot2 (v3.5.1).

A targeted comparative analysis was then conducted to assess the distribution of GLP-1R+ versus GLP-1R- cells across annotated cell types. GLP-1R expression status was determined at the single-cell level based on transcript detection above background threshold (GLP-1R transcripts > 0). Proportions of GLP-1R+ cells were calculated within each cell type and compared between GLP-1 exposed and non-exposed patient groups. Differences were summarized using descriptive metrics such as mean cell type frequencies, standard deviations, and percent differences per group.

#### Spatial Transcriptomics Differential Expression Analysis

Differential expression analysis was performed on cancer (malignant) cells and macrophages. To ensure balanced comparisons, only one sample per patient was included; when both pre- and post-treatment samples were available, the post-treatment sample was retained. This resulted in a comparison of 7 exposed (2 pre and 5 post-treatment samples) versus 4 non-exposed (4 post-treatment) samples. Differentially expressed genes (DEGs) were identified using the Wilcoxon rank-sum test implemented in Seurat’s *FindMarkers* function, and results were visualized as volcano plots using the Enhanced Volcano package (v1.13.2). A total of 894 out of 5,000 genes met significance criteria (adjusted p<0.05, |log_2_ fold change| > 0.5). The top 30 DEGs from each group were further subjected to pathway enrichment analysis using Enricher with the MSigDB Hallmark 2020 gene sets.

#### Spatial Neighborhood Analysis

To investigate the spatial proximity of different cell types, we utilized the SPACEc package (v0.1.2)^23^. The dataset was subdivided into two groups: (i) GLP-1 exposed/GLP-1R+ cells, (ii) GLP-1 exposed/GLP-1R- cells, (iii) GLP-1 non-exposed/GLP-1R+ cells, and (iv) GLP-1 non-exposed/GLP-1R- cells. Cellular neighborhoods (CNs) were identified using k = 6, as determined by the Elbow diagnostic. Each CN was then annotated based on the average log2 fold change (log2FC) expression of cell type markers. To visualize the spatial arrangement and associations between distinct CNs, we applied sp.pl.cn_map(). This function generates a hierarchical spatial context map, illustrating the co-occurrence and spatial organization of different CNs within the tissue. Associations were determined based on the frequencies of local CN combinations, where, for example, a total frequency of 10% for CN 1 and CN 2 in the spatial context map indicates that their co-occurrence locally accounts for 10% of the spatial regions across the tissue. This approach provides insights into the tissue’s spatial microarchitecture and highlights prominent cellular interactions and CN compositions. Infiltration was quantified by counting non-malignant cells within a 100 μm^2^ radius around each malignant cell centroid, based on transcript-defined cell boundaries from CosMx segmentation data.

### Retrospective Clinical Cohort

This multicenter, retrospective cohort study analyzed patients with stage I–III TNBC who received neoadjuvant treatment with the KEYNOTE-522 regimen (pembrolizumab plus chemotherapy) at two academic institutions (UT Southwestern Medical Center (UTSW) and its affiliate health system, Parkland Health, and City of Hope) between July 2021 and December 2023. Patients were identified through institutional tumor registries and electronic pharmacy records. Eligible participants were adults (≥18 years) with histologically confirmed TNBC (estrogen and progesterone receptor expression ≤10%; HER2-negative by ASCO/CAP guidelines) who completed at least four cycles of chemoimmunotherapy. The exposed cohort consisted of patients with documented continuous use of GLP-1RA or DPP4i from neoadjuvant treatment initiation through completion, verified via prescription records and clinical documentation. Two comparator groups were established: 1) patients with type 2 diabetes using non-GLP-1-based glucose-lowering agents and 2) non-diabetic controls. Key exclusion criteria included discontinuation or initiation of GLP-1 targeting agents during treatment, prior malignancies within five years, incomplete neoadjuvant treatment, or missing pathological response data. The institutional review boards of all participating centers approved the study.

Data were collected using standardized electronic case report forms from electronic health records, pharmacy databases, and pathology reports. Variables included demographic characteristics, clinical parameters (body mass index, diabetes duration, comorbidities), tumor characteristics (stage, grade, Ki-67 index), treatment details, and concomitant medications. Medication adherence was confirmed through pharmacy dispensing records requiring ≥2 filled prescriptions during treatment, supplemented by clinician documentation. The primary outcome was pathological complete response (pCR), defined as ypT0/Tis ypN0 upon pathology review by expert breast pathologists at each institution.

Analyses were performed using R version 4.3.1. Continuous variables were summarized as mean ± standard deviation or median with interquartile range and compared using t-tests/ANOVA or Mann–Whitney U/Kruskal–Wallis tests, as appropriate. Categorical variables were analyzed using χ^2^ or Fisher’s exact tests, with Bonferroni correction for multiple comparisons. To address confounding, we performed 1:1 propensity score matching using logistic regression with four covariates: age, body mass index, diabetes medication burden, and tumor stage^24,25^. Nearest-neighbor matching with a caliper width of 0.2 standard deviations was employed. Covariate balance was assessed via standardized mean differences (<0.1 indicated adequate balance). The primary analysis compared pCR rates between exposure groups using logistic regression in both the full cohort and propensity-matched subsets. Exact p-values are reported throughout.

## Results

### GLP-1 signaling stimulates growth and survival in TNBC cell lines

**Figure 1:**
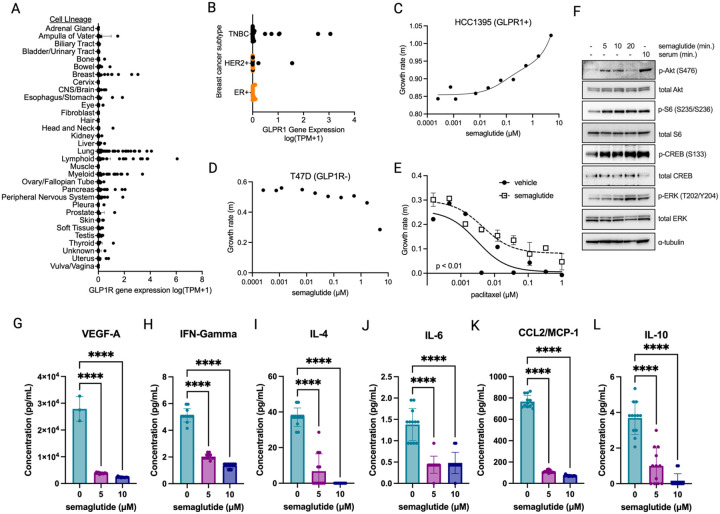
GLP-1R expression in cell lines determines sensitivity to GLP-1 stimulation. **A.** Pan-cancer DepMap analysis of GLP-1R transcript expression levels in all cell lines (n=1684). **B.** GLP-1R transcript expression levels in DepMap breast cancer cell lines stratified by breast cancer sub-type. Orange=ER+ cell lines, Black=ER-negative cell lines. N=70. **C.** Growth rate of HCC1395 TNBC cell line (GLP-1R+) in response to semaglutide for 3 days. 3 replicates of each dose point were used to calculate growth rates by linear regression, followed by nonlinear fit (R^2^=0.97). **D.** Growth rate of T47D (GLP-1R-negative) cell lines in response to semaglutide for 3 days, (n=3 replicates per dose point). **E.** Growth rate of HCC1395 cells co-treated with semaglutide (2.5 – 5 μM) versus vehicle control and increasing doses of paclitaxel. N=3 replicates per dose point. Semaglutide 2.5 and 5 μM doses are shown as combined mean ± SD. Data is fit by nonlinear regression and compared by Extra sum of squares F test. **F.** HCC1395 cells were serum starved, then stimulated with semaglutide or serum for the times indicated and analyzed by SDS-PAGE and immunoblot analysis with the indicated antibodies. See also Supplementary Fig. S1. **G-L.** HCC1395 cells were grown in indicated doses of semaglutide versus vehicle control for 3 days then cytokine levels were measured by ELISA. N=12 replicates per group. **** p<0.0001 by one-way ANOVA and Dunnett’s multiple comparison test. See also Supplementary Fig. S1 and S2.

We hypothesized that the GLP-1R would be expressed in patient-derived cell lines, which could serve as models for mechanistic studies. First, to define the spectrum of GLP-1R expression in a pan-cancer analysis the human Dependency Map (DepMap) cell lines (n=1684) were interrogated for GLP-1R transcript levels. GLP-1R transcripts were detected in 664 cell lines (39.4%) and undetectable in the rest (Fig. 1A). Certain tumor lineages had a higher proportion of GLP-1R+ cell lines including pancreas (63.6%, n=55), myeloid (60.4%, n=73), peripheral nervous system (60%, n=45), lung (54.9%, n=213), breast (51.4%, n= 70), and lymphoid (37.9%, n=187). Other tumor lineages had uniformly low or undetectable GLP-1R expression levels, including adrenal gland, biliary tract, bladder & urinary tract, cervix, kidney, muscle, skin, soft tissue, etc. Thus, GLP-1R can be expressed in a significant proportion of solid and liquid tumor patient-derived cell lines.

To better understand the heterogeneity of GLP-1R within a specific tumor histology, we more carefully examined the breast cancer subtypes. The breast cancer cell lines were stratified by the three predominant subtypes (ER+, HER2+ and TNBC) and GLP-1R transcript expression was interrogated. This showed that GLP-1R transcripts were detected in a subset of TNBC and HER2+ cell lines, but not in ER+/HER2- cell lines (Fig. 1B Fisher’s exact p= 0.16). Notably, in the HER2+ sub-group, only the cell lines lacking ER had significant GLP-1R transcript levels detected compared to ER+/HER2+ patients in this cohort (Fig. 1B). Considered together there was a significant enrichment for GLP-1R expression in ER-negative compared to ER+ cell lines (P = 0.017 by Fisher’s exact). To more carefully define robust GLP-1R expression in breast cancer cells we focused on cell lines with log (TPM+1) > 0.2. There were 6 TNBC cell lines that met this criterion (HCC1187, HCC1395, SUM149PT, COLO824, HCC1143, HCC70), comprising 18% of the TNBC cell lines analyzed. Two HER2+ cell lines met this criterion (UACC3199, HCC2218). Taken together, this analysis identifies robust GLP-1R expression in a subset of ER-negative, but not ER+, breast cancer cell lines.

To determine if GLP-1R expression is sufficient to confer sensitivity to GLP-1 agonists we obtained representative GLP-1R+ and GLP-1R- breast cancer cell lines. The TNBC HCC1395 cell line had one of the highest GLP-1R transcript levels (log (TPM+1) =2.57). When this cell line was exposed to increasing doses of semaglutide, we observed increased growth rates (Fig. 1C). In contrast, the ER+ cell line T47D had undetected GLP-1R levels and was unaffected by semaglutide treatment (Fig. 1D). A third TNBC cell line with strong GLP-1R expression (HCC1187, log (TPM+1) =3.06) also evinced increased growth rates due to semaglutide stimulation (Supplementary Fig. S1). Taken together, these data suggest that GLP-1R expression confers sensitivity to GLP-1 agonists in TNBC cell lines.

Since GLP-1RA treatment stimulated proliferation, we also tested chemotherapy sensitivity in cell lines. The GLP-1R+ TNBC cell line HCC1395 was treated with semaglutide and exposed to increasing doses of paclitaxel, a critical component of curative therapy for TNBC. Semaglutide treatment caused increased cell growth compared to vehicle-treated cells exposed to paclitaxel (Fig. 1E, p<0.01). These data suggest semaglutide could antagonize the antitumor action of chemotherapy.

Resistance to chemotherapy is often linked to aberrant cell signaling in cancer cells. The GLP-1R is a G-protein coupled receptor (GPCR) that signals through activation of G-proteins, stimulating adenylyl cyclase to increase cyclic AMP (cAMP) levels resulting in PKA activation.^26^ GPCR βγ subunits can also directly activate signaling pathways including the phosphoinositide 3-kinase (PI3K) and protein kinase B (Akt). We tested whether GLP-1 signaling through the GLP-1R can also activate these pathways in TNBC cells. Short-term treatment with semaglutide stimulated kinase cascades including phosphorylation of Akt, S6, CREB and ERK in GLP-1R+ TNBC cells (Fig. 1F). Stimulation with semaglutide led to phosphorylation levels equivalent to serum stimulation (Fig. 1F). Thus, growth-inducing signaling cascades are stimulated by GLP-1 agonists in breast cancer cell lines.

Cancer cells commonly elaborate cytokines to signal to immune cells. Given the alterations in cell signaling observed in GLP-1 exposed tumors we hypothesized that GLP-1 treatment could affect cytokine secretion from GLP-1R+ tumor cells. To test this hypothesis, we exposed GLP-1R+ HCC1395 TNBC cells to semaglutide and measured cytokine levels in tissue culture media. This showed that 6 cytokine levels were decreased by semaglutide treatment (VEGF-A, IFN-γ, IL-4, IL-6, CCL2/MCP-1, IL-10; Fig. 1G–L). In contrast, other cytokine levels were unaffected (IL-13, GM-CSF, IL-12/IL-23 p40, IL18/IL-1F4; Supplementary Fig.S2). Therefore, GLP-1 stimulation causes significant decreases in a subset of cytokines, including those important for anti-tumor immunity (e.g. IFN- γ).

### Identification of GLP-1R Expression in TNBC Specimens

**Figure 2:**
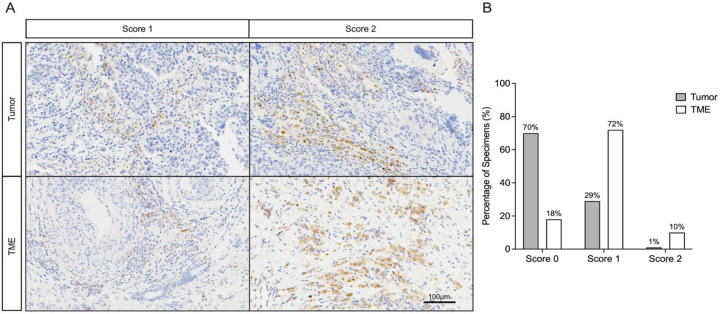
Immunohistochemical Analysis of GLP-1R Expression in TNBC Specimens. **A.** Representative IHC staining for GLP-1R. Tumor score 1: Weak membranous staining in glandular structures. Tumor score 2: Clusters of tumor cells with strong membranous staining. TME score 1: Sparse stromal staining in fibroblasts. TME score 2: Intense staining in immune cell clusters consistent with macrophages and lymphocytes. **B.** Prevalence of GLP-1R Scores in TNBC Specimens (N=100).

We next sought to characterize GLP-1R in human TNBCs by immunohistochemical analysis of 100 primary tumor specimens. There were distinct GLP-1R expression patterns between tumor cells and the TME. Whereas 70% showed no GLP-1R expression in tumor cells (score 0), there was 29% with weak to moderate positivity (score 1), and only 1% demonstrated strong, clustered membranous staining (score 2). Conversely, the TME displayed widespread positivity: 72% scored 1 characterized by sparse, weak staining in stromal fibroblasts, endothelial cells, and immune cells such as macrophages and lymphocytes, frequently distributed near fibrovascular regions; 10% of specimens showed strong, clustered staining (score 2), which localized to intense immune cell aggregates and fibroblasts, predominantly within peritumoral stromal regions and marked by pronounced heterogeneity in immune-rich zones (Fig. 2A and B). Thus, GLP-1R was detected in both tumor cells and TME cells in TNBCs, although it was more prevalent in the TME.

We more carefully determined the balance of GLP-1R in various tumor cell types. There was a discordance in GLP-1R expression between tumor cells and the TME. In most cases (n=52) the TME was positive whereas tumor cell staining was absent (score 0). Among cases with low tumor cell staining (score 1, n=29), all showed concurrent TME positivity. A small fraction of cases (n=18) was negative for both TME and tumor cell staining. Therefore, GLP-1R+ tumor cell staining was always found with GLP-1R+ TME, but some tumors had only TME staining.

### GLP-1R+ cell types in TNBCs identified by spatial transcriptomics

**Figure 3:**
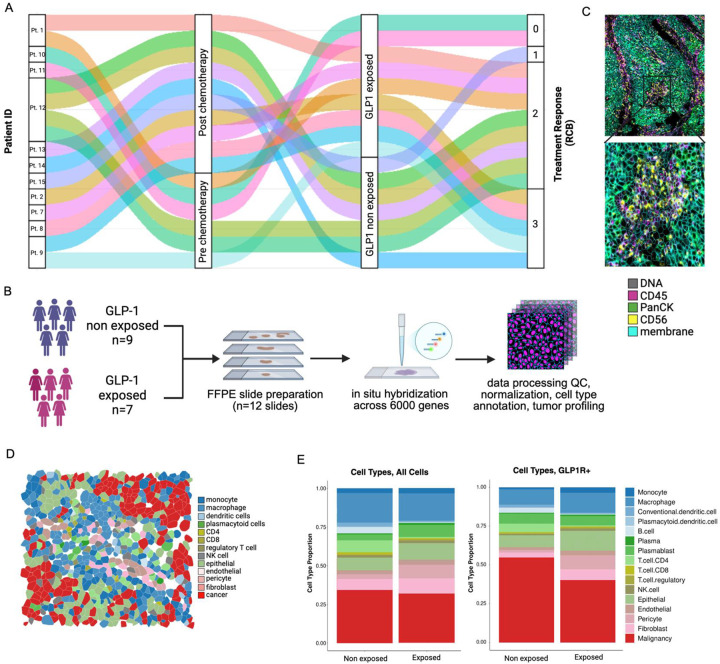
Altered gene expression in GLP-1 targeting agent exposed (GLP-1 exposed) malignant cells. **A.** Sankey plot representing the flow of patient samples within the cohort. Color code highlights different samples (n=16). **B.** Experimental workflow for spatial analysis of tumor specimens. **C.** Zoomed-in region of interest of a representative sample showing the fluorescence channels (DNA, CD45, PanCK, CD56, membrane) used for cell segmentation, along with cell boundaries. **D.** Cell phenotyping and spatial representation of the same ROI from C displayed as cell polygons colored by cell types identified. **E.** Proportion barplots of cell types separated by patients GLP-1 exposed and GLP-1 non-exposed in all cells and GLP-1R+ cells (n=16).

To further investigate the spatial distribution and cellular context of GLP-1R expression in TNBC, we performed high-plex spatial RNA profiling using the CosMx Spatial Molecular Imager platform (Fig. 3A and B). This approach enabled direct visualization of GLP-1R transcripts (GLP-1R+ cells) within tumor tissue and identification of the specific cell populations expressing them. We included pre-treatment and post-treatment tumor specimens from diabetic patients exposed to GLP-1 targeting agents (GLP-1 exposed; n=7) and diabetic patients non-exposed to GLP-1 targeting agents (GLP-1 non-exposed; n=4) but otherwise matched for other clinical variables (Fig. 3A; Supplementary Table S1). Overall, both GLP-1 exposed and non-exposed patients exhibited a heterogeneous mixture of cell types within their tumors. Quantification of major cellular compartments (Fig. 3C and D) revealed comparable proportions of immune, stromal and epithelial cell types between the GLP-1 exposed and non-exposed groups (Fig. 3E). Also, there was no significant cell type–specific enrichment of GLP-1R+ cells across the cohort, with comparable proportions of GLP-1R+ cells detected within each major cell type (GLP-1R+: mean = 4.02%, SE = 0.24%, range = 2.01–8.42%; Supplementary Fig. S3). Therefore, GLP-1R+ cells were broadly detected in malignant and non-malignant cells in all tumors.

### GLP-1 exposure alters tumor-immune interactions in TNBC

**Figure 4:**
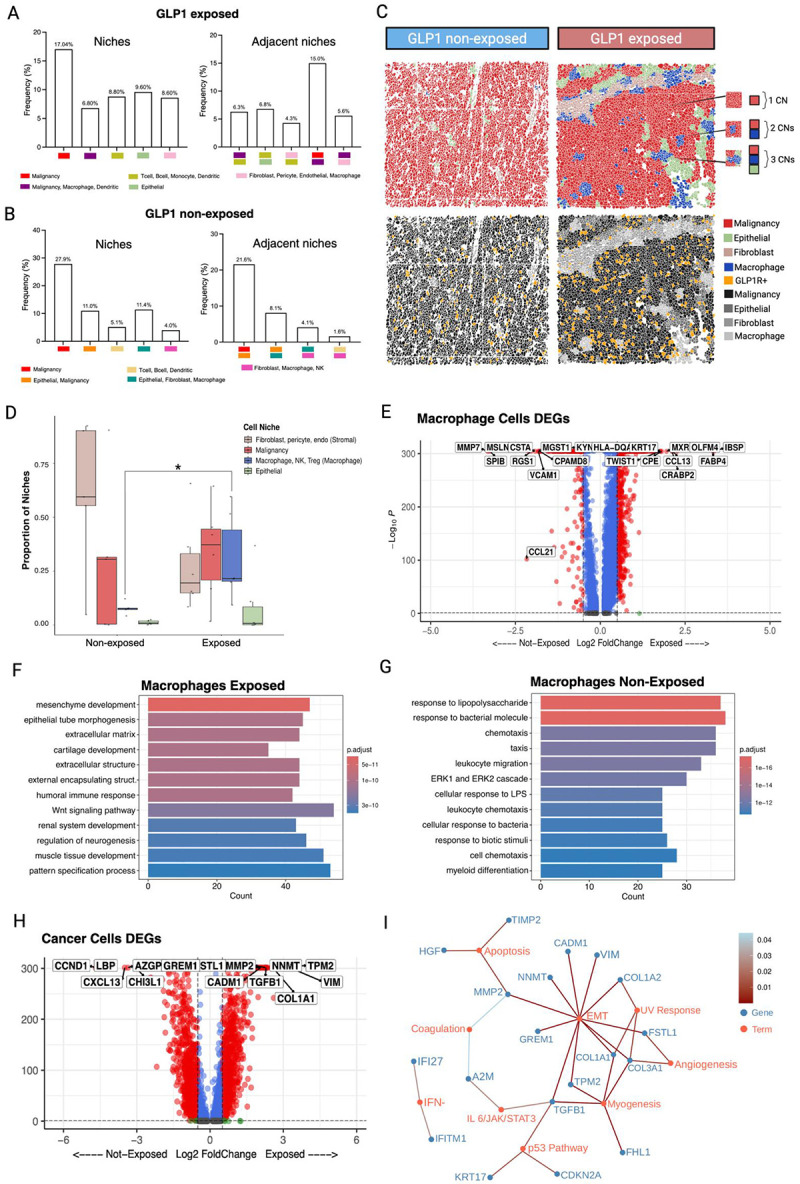
GLP-1 targeting agent exposure alters tumor-immune interactions in TNBC. **A.** Bar plots showing the relative frequency of major spatial niche classes identified in GLP-1 exposed tumors. (Right) Corresponding frequencies of each niche occurring in direct spatial proximity to GLP-1R+ cells. **B**. Equivalent analysis for GLP-1 non-exposed tumors, highlighting differences in niche composition and adjacency patterns relative to GLP-1R+ populations. Colors denote the dominant cell types comprising each niche. **C.** ROI representation of niches as spatial plots. Spatial analysis showing three representative cellular niches (CN) composed of 1) malignant cells only; 2) malignant cells + myeloid cells; 3) malignant cells + myeloid cells + epithelial cells. Bottom panels highlight GLP-1R+ cells in orange. **D.** Proportion box plots showing the distribution of spatial niches across GLP-1–exposed and non-exposed tumors (n=11). Each dot represents one sample; boxes indicate the median and interquartile range. *p<0.05 by Wilcoxon rank-sum. **E.** Volcano plot showing differentially expressed genes (top 10 genes by log Fold Change and adjusted p-value labeled) between GLP-1 non-exposed and GLP-1 exposed macrophages cells (n=11). **F.** GSEA (gene set enrichment analysis) of pathways upregulated in GLP-1 exposed tumors. **G.** GSEA of pathways upregulated in GLP-1 non-exposed tumors. **H.** Volcano plot showing differentially expressed genes (top 10 genes by log Fold Change and adjusted p-value labeled) between GLP-1 non-exposed and GLP-1 exposed malignant cells (n=11). **I**. Network plot of the most enriched biological pathways derived from differentially expressed genes in malignant cells exposed to GLP-1.

Since we detected GLP-1R in both malignant and non-malignant cells, we next performed further analysis to assess how GLP-1 targeting agent exposure altered the spatial properties of malignant cells with immune cells. Tumors were stratified into two groups: GLP-1 exposed and GLP-1 non-exposed. Then the local cellular neighborhoods surrounding GLP-1R+ cells were quantified to identify dominant immune and stromal niche architectures. In GLP-1 exposed tumors, malignant cells showed markedly increased spatial co-localization with macrophages, with cancer–macrophage niches accounting for 15% of all GLP-1R+ neighborhoods (Fig. 4A). In contrast, in non-exposed tumors, the predominant niche adjacent to GLP-1R+ cells were epithelial–malignant combinations (21.6%) (Fig. 4B). Together, these results demonstrate that GLP-1 exposure alters the spatial arrangement of cellular neighborhoods within the tumor microenvironment, repositioning GLP-1R+ cells into macrophage-dense and stromal-rich regions.

Next, we assessed if macrophage spatial differences could be explained by niche proportions. Tumors were segmented into four separate niches comprising: 1) malignant cells; 2) epithelial cells; 3) stroma (pericytes, fibroblasts, endothelia); 4) macrophages (that included a small proportion of associated NK and Tregs) (Fig. 4C). We observed that the relative proportion of macrophage-enriched niches was significantly higher in GLP-1 exposed samples compared to non-exposed counterparts (Wilcoxon rank-sum p=0.0087; Fig. 4D). In contrast, no significant differences were observed in stromal (fibroblast, pericyte, endothelial), malignant, or epithelial niche proportions (p>0.24 for all) (Fig. 4D). Thus, macrophage-predominant niches were specifically associated with GLP-1 exposure.

Deeper analysis of macrophage gene expression was performed to understand these spatial differences. We found that GLP-1 exposure increased genes associated with M2-skewed polarization in tumor-associated macrophages. Differential expression analysis identified a signature in GLP-1 exposed tumors defined by upregulation of pro-fibrotic and immunomodulatory genes (TWIST1, CCL13, FABP4), consistent with a pro-tumoral phenotype. Conversely, macrophages from non-exposed tumors maintained a balanced M1/M2 profile with features of immune interaction (VCAM1) and matrix remodeling (MMP7) (Fig. 4E). Pathway analysis revealed that GLP-1 targeting agent exposure induces a fundamental reprogramming of tumor-associated macrophages. Macrophages from exposed tumors demonstrated a pro-fibrotic and mesenchymal transformation, characterized by extracellular matrix organization and mesenchyme development pathways (Fig. 4F). We also detected pro-tumorigenic Wnt signaling^27^ and tissue morphogenesis pathways, suggesting an immunosuppressive, wound-healing microenvironment.^28^ In contrast, non-exposed macrophages maintained immunostimulatory pathways including activated LPS response, preserved capacity for immune cell recruitment via intact leukocyte migration and chemotaxis signaling, and functional inflammatory activation through ERK1/2 cascade signaling^29^ (Fig. 4G).

Since macrophage niches were associated with malignant cells in GLP-1 exposed tumors we also examined malignant cell gene expression differences. Malignant cells from GLP-1 exposed patients displayed a more pronounced epithelial-to-mesenchymal transition (EMT) phenotype compared to those from non-exposed tumors (Fig. 4H and I; Supplementary Table S1). Differential expression analysis identified upregulation of mesenchymal markers (COL1A1, GREM1, COL3A1, NNMT, TGFB1, COL1A2, CADM1, TPM2, MMP2, VIM, FSTL1, logFC > 2, range = 2.11–3.16; padj < 0.01) and downregulation of epithelial genes (CCND1, LBP, CXCL13, CALML5, AZGP1, S100P, CHI3L1, CTTN, SPIB, KRT15, logFC < 3.37, range = 4.50–2.80; padj < 0.01) in GLP-1 exposed malignant cells compared to non-exposed patients (Fig. 4H and I). Other enriched upregulated pathways in the GLP-1 exposed tumors included p53 response, interferon alpha, IL6/Jak/STAT signaling, and others. Thus, GLP-1 exposure induced specific transcriptional changes in malignant cells, which could be partially due to their association with immunosuppressive macrophages in the TME.

### Patients taking GLP-1 therapies have decreased response rates

**Figure 5:**
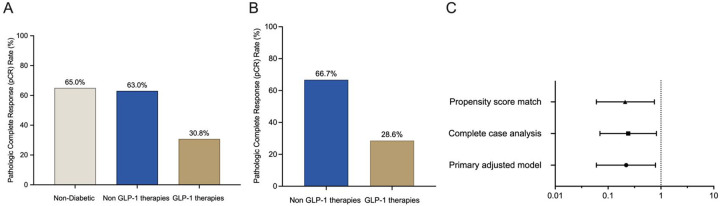
pCR rate among patients with TNBC treated with neoadjuvant chemoimmunotherapy. **A.** pCR in patients stratified by GLP-1 exposure and diabetic status (n=343). **B.** pCR post-PSM in diabetic patients stratified by GLP-1 exposure. **C.** Forest plot displaying the adjusted odds ratios (ORs) and 95% confidence intervals (CIs) for achieving pCR among patients with early-stage triple-negative breast cancer receiving the KEYNOTE-522 regimen, comparing those using GLP-1 targeting agents versus non GLP-1 antidiabetic therapies. The odds ratios are derived from three statistical models: a primary multivariable-adjusted logistic regression model (OR, 0.22; 95% CI, 0.06–0.79), a complete case analysis (OR, 0.24; 95% CI, 0.07–0.82), and a propensity score–matched analysis (OR, 0.21; 95% CI, 0.06–0.75). GLP-1 targeting agent use was associated with significantly lower odds of pCR across all analyses.

The data thus far indicates that GLP-1 exposure induces reprograms tumor cell and macrophage biology in ways that could be detrimental to therapeutic responses. This led us to examine whether patients exposed to GLP-1 targeting agents had worse treatment outcomes. We analyzed a retrospective, multicenter cohort study of 343 patients with early-stage TNBC treated with the KEYNOTE-522 neoadjuvant chemoimmunotherapy regimen for associations between diabetes medication use and pathologic complete response (pCR). Participants were categorized into three groups: non-users of diabetes medications (n=271), users of non-GLP-1-based antidiabetic therapies (n=46), and users of GLP-1 receptor agonists (GLP-1RA) or DPP4 inhibitors (DPP4i) (collectively termed GLP-1 targeting agents; n=26). The GLP-1 targeting agent cohort consisted of patients with verified continuous use from chemotherapy initiation through completion, with the majority (23/26, 88.5%) receiving GLP-1RAs and the remainder (4/26, 15.4%) on DPP4is. Combination therapy was common, with 53.8% concurrently taking metformin. A significant disparity in pCR rates was observed across groups (Fig. 5A). Patients receiving GLP-1 targeting agents exhibited markedly reduced pCR rates (30.8%) compared with non-diabetic patients (65%) and diabetic patients taking non-GLP-1-based therapies (63%) with a p<0.001 for overall comparison. The observed pCR rate of 65% in our non-diabetic cohort aligns with the 64.8% pCR rate reported in the KEYNOTE-522 trial’s pembrolizumab-chemotherapy arm. This result indicates that GLP-1 targeting agent users achieved significantly lower pCR rates than both trial benchmarks and our non-diabetic controls.

To investigate the basis of this discrepancy in pCR rates the baseline characteristics across groups were assessed (Table 1). Compared to the non-diabetic cohort, patients receiving antidiabetic therapies were older (p=0.005), had elevated body mass index (p<0.0001), demonstrated higher rates of hypertension (p<0.0001) and hyperlipidemia (p<0.0001). In contrast, comparisons between the non GLP-1 therapy cohort and the GLP-1 targeting agents cohort revealed no significant differences in age (median: 57.2 vs. 60.0 years, p=0.49), BMI (median: 30.8 vs. 34.9 kg/m^2^, p=0.16) or hypertension (65.3% vs. 84.6%; p=0.065). Tumor characteristics including histologic subtype, grade, Ki67 expression, and cancer stage were comparable between the two diabetes medication cohorts with no significant disparities (Supplementary Table S2).

To address potential confounding variables, propensity score matching (PSM; 1:1 ratio) was performed, balancing the cohorts for age, BMI, diabetes medication burden, and cancer stage (Table 2). After matching, the cohorts (GLP-1/DPP4 targeting agents vs. non-GLP-1 antidiabetic therapies) were evenly distributed (n=21 each). Covariates were well-balanced post-PSM: age differences were non-significant (p=0.87 vs. pre-PSM p=0.61), and BMI means were indistinguishable (34.8 vs. 34.5 kg/m^2^; p=0.87 vs. pre-PSM p=0.17). Cancer stage distributions were fully aligned post-matching (Stage I: 4.76%, Stage II: 76.19%, Stage III: 19.05%; p=1.00). Additionally, the number of diabetes medications per patient showed no significant imbalance post-PSM (p=0.56 vs. pre-PSM p=0.21), thus mitigating baseline disparities that could account for the difference in pCR outcome.

After covariate balancing through PSM, the disparity in pCR rates remained significant. Prior to matching, pCR was achieved in 63.0% of patients receiving non-GLP-1 antidiabetic therapies compared to 30.8% in the GLP-1/DPP4 targeting agents’ cohort (p=0.0085). Post-PSM analysis this significant divergence remained, with pCR rates of 66.7% (non-GLP-1 therapies) versus 28.6% (GLP-1/DPP4 targeting agents; p=0.0134; Fig. 5B). This persistent significant association suggests a robust inverse relationship between GLP-1 targeting agents and chemoimmunotherapy efficacy that is unlikely to be attributed to the balanced confounders.

Notable differences existed in concomitant diabetes therapies across pre-PSM groups. Among GLP-1 targeting agent users, 88.5% (23/26) received GLP-1RAs while 15.4% (4/26) used DPP4i. Combination therapy was common, with 53.8% (14/26) concurrently taking metformin and 38.5% (10/26) using insulin. The non-GLP-1 diabetes cohort showed higher metformin use (80.4% [37/46]) and insulin dependence (56.5% [26/46]). Despite these differences, the number of concurrent diabetes medications per patient was balanced between diabetes cohorts (median 2 medications in both groups, p=0.21), and adjustment for medication burden in propensity models did not attenuate the pCR association (adjusted OR 0.24, 95% CI 0.07–0.83).

The negative association between GLP-1 targeting agents and pCR was evident across multiple modeling approaches. In the primary adjusted model (including age, BMI, diabetes severity, and tumor stage), GLP-1 targeting agent use was associated with significantly reduced odds of achieving pCR (OR 0.22, 95% CI 0.06–0.79), equivalent to a 78% relative reduction in response likelihood. The consistency of this effect across sensitivity analyses including complete case analysis (OR 0.24, 95% CI 0.07–0.82), strengthens the evidence for an independent treatment-modifying effect. Notably, all confidence intervals excluded the null value (dashed line at OR=1), with the tightest precision observed in the propensity-matched analysis (OR 0.21, 95% CI 0.06–0.75) (Fig. 5C). Thus, the negative association between GLP-1 targeting agents and pCR was evident across all tested models.

## Discussion

This study identifies a previously underrecognized and clinically significant adverse interaction between GLP-1 targeting agents and the efficacy of neoadjuvant chemoimmunotherapy in TNBC. Our mechanistic investigations attribute GLP-1-induced treatment resistance to a dual axis of action. First, we provide direct evidence that GLP-1R is functionally active in a subset of TNBC tumors and cell lines. GLP-1 exposure in GLP-1R+ models stimulated key oncogenic signaling pathways (PI3K-AKT and MEK-ERK), enhanced cell proliferation, and induced resistance to paclitaxel. In patient tumors, this was reflected in an epithelial-to-mesenchymal transition signature in malignant cells from GLP-1 exposed patients, a well-established pathway associated with therapy resistance. Second, we demonstrate that GLP-1 signaling exerts immunomodulatory effects. Spatial transcriptomics revealed a reorganization of the TME, with GLP-1 exposed malignant cells residing in closer proximity to macrophages. Functionally, GLP-1 exposure transformed macrophages from immune-competent sentinels into pro-fibrotic effectors, enhancing extracellular matrix remodeling and mesenchymal transition while dampening key immune activation pathways. This phenotypic shift may establish an immunosuppressive niche that undermines anti-tumor immunity and confer resistance to chemo-immunotherapy.

Furthermore, our data reveal that patients using GLP-1RA or DPP4i while undergoing the KEYNOTE-522 regimen experienced a significant reduction in pCR rates, an effect that persisted after propensity score matching and multivariable adjustment. The magnitude of the observed clinical effect is substantial. The absolute reduction in pCR of over 30% in GLP-1 exposed patients translates to a 78% relative reduction in the odds of achieving a pCR. Given the established correlation between pCR and long-term survival outcomes in the context of KEYNOTE-522, this finding has implications for clinical decision-making. Crucially, the effect appears specific to GLP-1 targeting agents, as patients with diabetes on other glucose-lowering therapies had pCR rates equivalent to non-diabetic controls. This specificity, combined with the effective balancing of key confounders like BMI and diabetes severity in our propensity-matched analysis, suggests a direct, drug-mediated biological mechanism rather than a phenomenon driven by underlying metabolic conditions.^30,31^

Our findings further extend recent literature addressing the effect of GLP-1 therapies on cancer.^32^ While some epidemiological studies have suggested neutral or even protective effects, our data indicate that the context, specifically concurrent treatment with immunotherapy, is critical. The immunomodulatory properties of GLP-1 agents, often framed as anti-inflammatory, may be detrimental in the setting of cancer, where productive inflammation is a central component of effective therapy. The expression of GLP-1R we observed in the tumor stroma and immune cells provides a direct conduit for these therapies to influence the local immune environment. Furthermore, as we detect GLP-1R expression in a variety of solid and liquid malignancies these results may extend beyond the breast cancers examined here. Importantly, ER+ breast cancer cell lines were uniformly negative for GLP-1R, and immunotherapies are not currently approved for this subtype, suggesting that certain cancer subtypes could be safely treated with concurrent GLP-1RAs. However, a biomarker predictive of this different outcome as a function of cancer subtype remains to be established.^33^

Future efforts should focus on validating these findings in larger, prospective cohorts. Nevertheless, it is prudent to consider the clinical implications. As the use of GLP-1 targeting agents expands rapidly for diabetes, obesity, and other indications, a growing number of cancer patients will be exposed to these therapies. The significant metabolic benefits of these medications should be carefully weighed against their potential to compromise curative-intent cancer therapy. For patients with TNBC scheduled to receive chemoimmunotherapy, alternative diabetes management strategies may be preferable. Alternatively, targeting tumor-associated macrophages could be explored to abrogate the negative effects of GLP-1 targeting agents.^34^ In conclusion, this study unveils a critical clinical scenario that may significantly impact survival outcomes in an aggressive cancer, highlighting the imperative to more carefully assess how metabolic therapies impinge on cancer treatments.

## Supplementary Material

1

## Figures and Tables

**Table 1:** Baseline Characteristics of Patients by Cohort

	Non-diabetic (n=271)	Non GLP-1 therapies (n=46)	GLP-1 therapies (n=26)	P-Value
**Age, median [IQR]**	51.5 [44.1–63.7]	57.2 [48.5–64.7]	60 [50.0–67.0]	0.005
**Gender, n (%)**				0.99
Female	271 (100)	46 (100)	25 (96.2)	
Male	0 (0.0)	0 (0.0)	1 (3.8)	
**BMI, median (kg/m** ^**2**^ **) [IQR]**	28.2 [24.8–33.5]	30.8 [26.6–36.3]	34.9 [29.0–39.3]	<0.0001
**Race, n (%)**				0.021
Asian	27 (10.0)	5 (10.8)	5 (19.2)	
Black	46 (17.0)	10 (21.7)	9 (34.6)	
White	189 (69.7)	27 (58.7)	12 (46.2)	
Unknown	9 (3.3)	4 (8.7)	0 (0.0)	
**Ethnicity, n (%)**				0.35
Hispanic/Latino	83 (30.6)	19 (41.3)	8 (30.7)	
Not Hispanic/Latino	181 (66.7)	25 (54.3)	18 (69.3)	
Unknown	7 (2.6)	2 (4.3)	0 (0.0)	
**Menopausal status, n (%)**				0.004
Pre	134 (49.4)	15 (32.6)	5 (19.2)	
Pos	131 (48.3)	31 (67.4)	20 (77.0)	
Peri	5 (1.9)	0 (0.0)	0 (0.0)	
N/A^a^/Unknown	1 (0.4)	0 (0.0)	1 (3.8)	
**Hypertension, n (%)**				<0.0001
Yes	60 (22.0)	30 (65.3)	22 (84.6)	
No	211 (78.0)	16 (34.7)	4 (15.4)	
**Hyperlipidemia, n (%)**				<0.0001
Yes	65 (24.0)	29 (63.0)	19 (73.0)	
No	206 (76.0)	17 (37.0)	7 (27.0)	
**Type 2 DM, n (%)**				<0.0001
Yes	3 (1.1)	46 (100)	23 (88.4)	
No	268 (98.9)	0 (0.0)	3 (11.6)	
**Histology, n (%)**				0.015
Ductal	249 (91.9)	35 (76.0)	23 (88.5)	
Lobular	4 (1.5)	1 (2.2)	0 (0.0)	
Unspecified/Other^b^	18 (6.6)	10 (21.8)	3 (11.5)	
**Tumor Grade, n (%)**				0.15
1	1 (0.4)	1 (2.2)	0 (0.0)	
2	32 (11.8)	6 (13.0)	5 (19.3)	
3	234 (86.3)	38 (82.6)	19 (73.0)	
Unknown	4 (1.5)	1 (2.2)	2 (7.7)	
**Ki67%, median [IQR]**	80 [70–90]	72 [50–85]	70 [45–83]	<0.0001
**Clinical T stage, n (%)**				0.72
1	32 (11.8)	3 (6.5)	5 (19.3)	
2	159 (58.7)	32 (69.6)	15 (57.6)	
3	55 (20.3)	7 (15.2)	4 (15.4)	
4	21 (7.8)	3 (6.5)	2 (7.7)	
X	3 (1.1)	1 (2.2)	0 (0.0)	
**Clinical N stage, n (%)**				0.02
0	136 (50.2)	25 (54.3)	14 (53.9)	
1	109 (40.2)	11 (24.0)	9 (34.6)	
2	9 (3.3)	6 (13.0)	0 (0.0)	
3	15 (5.6)	3 (6.5)	1 (3.8)	
X	2 (0.7)	1 (2.2)	2 (7.7)	
**DM/obesity treatment, n (%)** ^c^				
GLP-1 RA	0 (0.0)	0 (0.0)	23 (88.5)	
Metformin	0 (0.0)	37 (80.4)	14 (53.8)	
Insulin	0 (0.0)	26 (56.5)	10 (38.5)	
SFU	0 (0.0)	6 (13.0)	2 (7.7)	
SGLT2	0 (0.0)	10 (21.7)	2 (7.7)	
DPP4i	0 (0.0)	0 (0.0)	4 (15.4)	
Glitazones	0 (0.0)	6 (13.0)	0 (0.0)	
**Number of DM meds, n (%)**				<0.001
1	0 (0.0)	18 (39.1)	7 (26.9)	
2	0 (0.0)	19 (41.3)	11 (42.3)	
3	0 (0.0)	7 (15.2)	6 (23.1)	
4	0 (0.0)	2 (4.3)	2 (7.7)	

Abbreviations: DM: diabetes mellitus; SFU, Sulfonylureas; SGLT2, Sodium-Glucose Transport Protein 2 Inhibitors

a Menopausal status: “N/A” applied to the male patient in “GLP-1 therapies”

b Histology: “Other” includes non-ductal/lobular subtypes (e.g., micropapillary, papillary).

c Diabetes/obesity treatment: Multiple meds per patient allowed. Percentages sum to >100% for treatment types.

**Table 2.** Demographic and Clinical Characteristics Before Propensity Score Matching, and 1:1 Propensity score and exact-matched cohorts

	Before Propensity Score Matching				1:1 Propensity Score Matching			
	Non GLP-1 therapies (N=46)	GLP-1 therapies (N=26)	All patients (N=72)	P-Value	Non GLP-1 therapies (N=21)	GLP-1 therapies (N=21)	All patients (N=42)	P-Value
**Age, mean ± SD**	56.4±10.9	57.8±11.5	56.9±11.0	0.611	56.9±11.4	57.4±11.2	57.2±11.2	0.871
**Age, median [IQR]**	57.2 [48.5–64.7]	60 [50.0–67.0]	58 [48.7–65.0]	0.485	58 [51.0–64.8]	60 [53.0–66.0]	60 [51.0–66.0]	0.762
**BMI, mean ± SD**	31.7±7.2	34.2±7.9	32.6±7.5	0.173	34.8±7.6	34.4±6.7	34.6±7.0	0.869
**BMI, median [IQR]**	30.8 [26.6–36.3]	34.9 [29.0–39.3]	31.1 [27.4–37.2]	0.164	35.1 [30.4–37.0]	34.7 [29.3–39.0]	34.9 [29.9–39.0]	0.979
**Number of DM meds, mean ± SD**	1.84±0.84	2.11±0.90	1.94±0.87	0.212	1.95±0.74	2.09±0.83	2.02±0.78	0.559
**Number of DM meds, median [IQR]**	2 [1–2]	2 [1–3]	2 [1–2]	0.211	2 [1–2]	2 [2–3]	2 [1–3]	0.635
**Stage, n (%)**				0.192				1.00
I	1 (2.2)	3 (12.5)	4 (5.8)		1 (4.8)	1 (4.8)	2 (4.8)	
II	33 (73.3)	17 (70.8)	50 (72.5)		16 (76.2)	16 (76.2)	32 (76.2)	
III	11 (24.4)	4 (16.7)	15 (21.7)		4 (19.0)	4 (19.0)	8 (19.0)	
**pCR rate, n (%)**				0.008				0.013
No	17 (37.0)	18 (69.2)	35 (48.6)		7 (33.3)	15 (71.4)	22 (52.4)	
Yes	29 (63.0)	8 (30.8)	37 (51.4)		14 (66.7)	6 (28.6)	20 (47.6)	

Abbreviations: BMI, body mass index; DM, diabetes mellitus

Propensity score matching and exact matching were performed from the cohorts non GLP-1 therapies and GLP-1 targeting agents
