# Supplementary material for: GLP-1 Targeting Agents Impair Chemoimmunotherapy Effectiveness in Triple-Negative Breast Cancer
Source: Res Sq. 2026 Jan 9:rs.3.rs-8380296. Preprint. [Version 1] doi: 10.21203/rs.3.rs-8380296/v1 (PMC12803343; doi:10.21203/rs.3.rs-8380296/v1)
Supplement: 1 [file NIHPPRS8380296V1-supplement.pdf]

Supplementary Table 1: Baseline Characteristics of Profiled Cases and Controls

Case	Cohort	Age	BMI	Comorbidities	Number DM meds	Stage	RCB
patient_1	GLP-1 exposed	60	37.5	HTN, HLD, DM	2	III	2
patient_2	GLP-1 exposed	44	29.3	HTN, HLD	1	II	3
patient_7	GLP-1 exposed	45	51.9	HTN, HLD	1	II	2
patient_8	DPP4i exposed	65	29.05	HTN, HLD, DM	2	II	3
patient_9	DPP4i exposed	54	28.71	HTN, HLD, DM	4	III	3
patient_10	GLP-1 exposed	48	30.34	HTN	1	II	0
patient_11	GLP-1 exposed	63	23.37	No	1	II	0
patient_12	Not exposed	68	30.47	HTN, HLD, DM	1	II	2
patient_13	Not exposed	78	20.9	HTN, DM	1	II	2
patient_14	Not exposed	43	27.44	DM	1	II	3
patient_15	Not exposed	56	38.06	HTN, DM	2	II	1

Abbreviations: GLP-1, Glucagon-Like Peptide-1; DPP4i, Dipeptidyl-peptidase-4 inhibitors; BMI, body mass index; HTN, Hypertension; HLD, Hyperlipidemia; DM, Diabetes Mellitus; RCB, Residual Cancer Burden

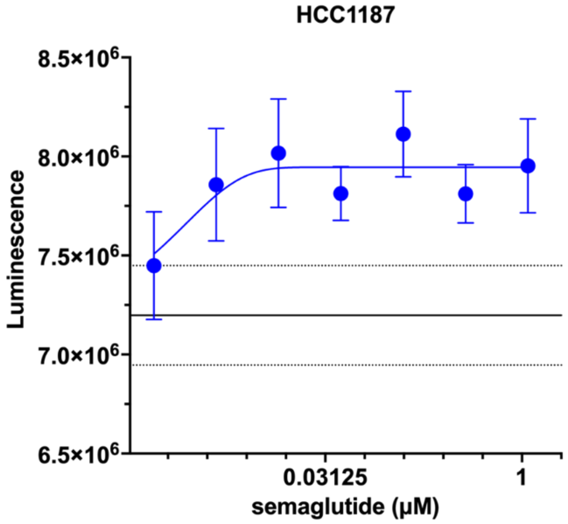

Supplementary Figure S1**: **Semaglutide dose–response of GLP-1R+ HCC1187 cells measured by CellTiter-Glo. *The GLP-1R+ cell line HCC1187 was grown in increasing doses of semaglutide and cell density was determined by cell titer glo. Data is mean +/- SEM of 4 replicates and representative of two independent experiments. Cell density (mean +/- SEM) of vehicle-treated control cells is depicted by horizontal solid and dotted lines.*

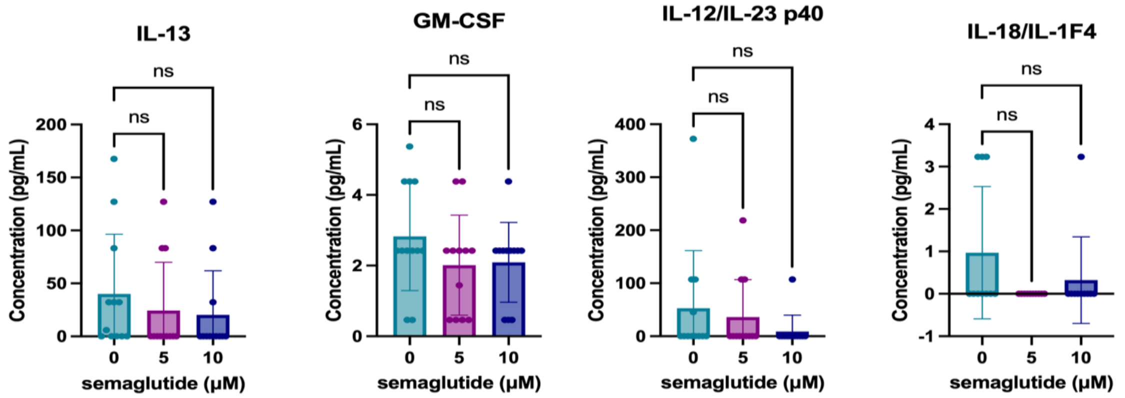

Supplementary Figure S2 (related to Fig. 1): Cytokines unaffected by GLP-1 stimulation. *Related to Fig. 5G-L. HCC1395 cells were grown in indicated doses of semaglutide versus vehicle control for 3 days then cytokine levels were measured by ELISA. n.s.=non-significant by one-way ANOVA and Dunnett's multiple comparison test.*

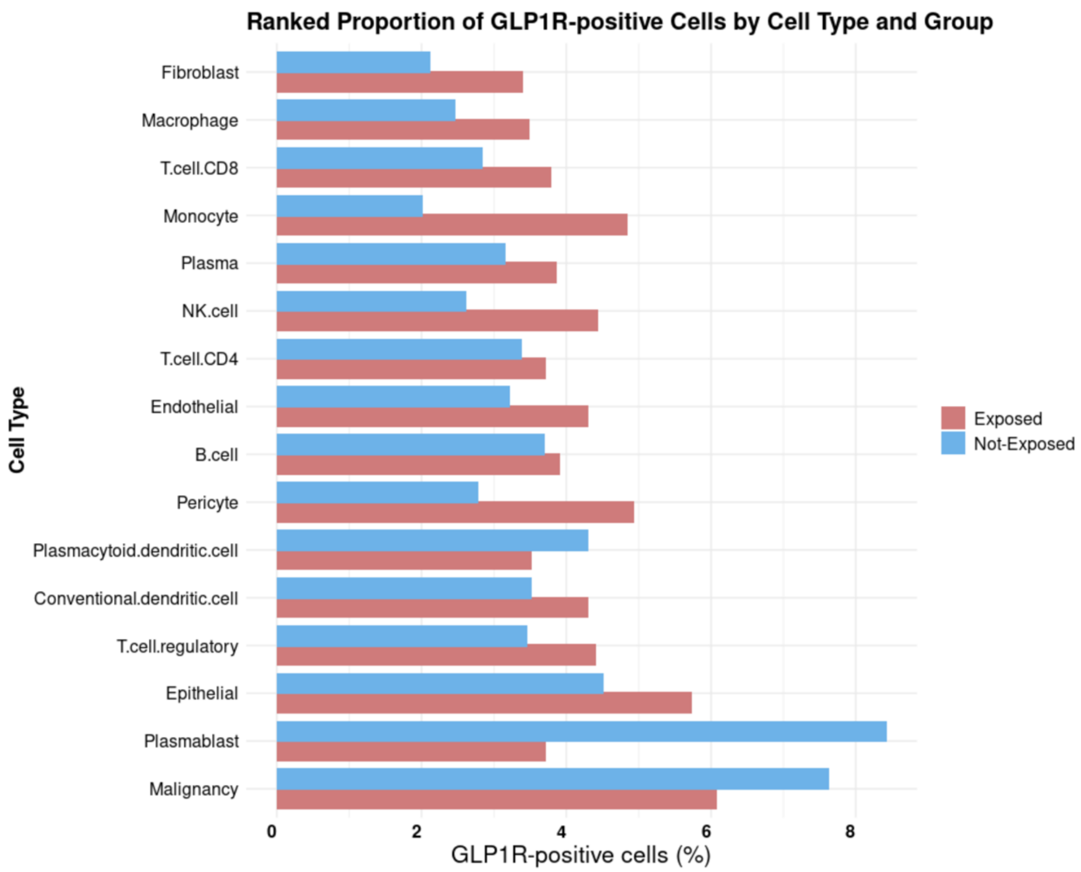

Supplementary Figure S3**: Proportion of GLP-1R+ cells across cell types in TNBC specimens stratified by GLP-1RA exposure.** *Spatial transcriptomic profiling was used to quantify the percentage of GLP-1R–expressing cells within each annotated cell type in TNBC tumors. Bars represent the mean proportion of GLP-1R-positive cells for GLP-1RA-exposed (red) and non-exposed (blue) cases. Cell types are ranked by their average GLP-1R positivity across groups.*

Supplementary Table 2: Baseline Characteristics of Patients by Diabetes Therapy Cohort

	Non GLP-1 therapies (n=46)	GLP-1 therapies (n=26)	P-Value
**Age, median [IQR]**	57.2 [48.5–64.7]	60 [50.0-67.0]	0.49
**Gender, n (%)**			0.33
Female	46 (100)	25 (96.2)	

Male	0 (0.0)	1 (3.8)	
**BMI, median (kg/m²) [IQR]**	30.8 [26.6–36.3]	34.9 [29.0–39.3]	0.16
**Race, n (%)**			0.35
Asian	5 (10.8)	5 (19.2)	
Black	10 (21.7)	9 (34.6)	
White	27 (58.7)	12 (46.2)	
Unknown	4 (8.7)	0 (0.0)	
**Ethnicity, n (%)**			0.37
Hispanic/Latino	19 (41.3)	8 (30.7)	
Not Hispanic/Latino	25 (54.3)	18 (69.3)	
Unknown	2 (4.3)	0 (0.0)	
**Menopausal status, n (%)**			0.66
Pre	15 (32.6)	5 (19.2)	
Pos	31 (67.4)	20 (77.0)	
Peri	0 (0.0)	0 (0.0)	
N/A[a]/Unknown	0 (0.0)	1 (3.8)	
**Hypertension, n (%)**			0.065
Yes	30 (65.3)	22 (84.6)	
No	16 (34.7)	4 (15.4)	
**Hyperlipidemia, n (%)**			0.31
Yes	29 (63.0)	19 (73.0)	
No	17 (37.0)	7 (27.0)	
**Type 2 DM, n (%)**			0.001
Yes	46 (100)	23 (88.4)	
No	0 (0.0)	3 (11.6)	
**Histology, n (%)**			0.35
Ductal	35 (76.0)	23 (88.5)	
Lobular	1 (2.2)	0 (0.0)	
Unspecified/Other[b]	10 (21.8)	3 (11.5)	
**Tumor Grade, n (%)**			0.5
1	1 (2.2)	0 (0.0)	
2	6 (13.0)	5 (19.3)	
3	38 (82.6)	19 (73.0)	

Unknown	1 (2.2)	2 (7.7)	
**Ki67%, median [IQR]**	72 [50–85]	70 [45–83]	0.71
**Stage**			0.49
IA	1 (2.2)	3 (11.5)	
IIA	24 (52.2)	11 (42.3)	
IIB	9 (19.6)	6 (23.0)	
IIIA	7 (15.2)	2 (7.7)	
IIIB	1 (2.2)	1 (3.8)	
IIIC	3 (6.5)	1 (3.8)	
X	1 (2.2)	2 (7.7)	
**DM/obesity treatment, n (%)**[c]			
GLP-1 RA	0 (0.0)	23 (88.5)	<0.001
Metformin	37 (80.4)	14 (53.8)	0.02
Insulin	26 (56.5)	10 (38.5)	0.12
SFU	6 (13.0)	2 (7.7)	0.72
SGLT2	10 (21.7)	2 (7.7)	0.14
DPP4i	0 (0.0)	4 (15.4)	0.02
Glitazones	6 (13.0)	0 (0.0)	0.08
**Number of DM meds, n (%)**			0.61
1	18 (39.1)	7 (26.9)	
2	19 (41.3)	11 (42.3)	
3	7 (15.2)	6 (23.1)	
4	2 (4.3)	2 (7.7)	
